# Model of a Device-Level Combined Wireless Network Based on NB-IoT and IEEE 802.15.4 Standards for Low-Power Applications in a Diverse IoT Framework

**DOI:** 10.3390/s21113718

**Published:** 2021-05-26

**Authors:** Juan Pablo García-Martín, Antonio Torralba

**Affiliations:** Electronic Engineering Department, Universidad de Sevilla, 41092 Seville, Spain; torralba@us.es

**Keywords:** wireless communications, low-power communications, IoT, combined wireless networks, NB-IoT, IEEE 802.15.4

## Abstract

With the development of the Internet of Things (IoT), Low Data Rate-Personal Area Networks (LR-WPAN) have been deployed for different applications. Now comes the need to integrate these networks in search of greater connectivity, performances, and geographic coverage. This integration is facilitated by the recent deployment of low power wide area networks (LPWAN) in the licensed bands, especially narrowband IoT (NB-IoT) and long-term evolution for machine-type communications (LTE-M), which are standardized technologies that will continue evolving as part of the fifth generation (5G) specifications. This paper proposes a design methodology for combined networks using LR-WPAN and LPWAN technologies. These networks are combined at the device level using a cluster-tree topology. An example is shown here, where an existing IEEE 802.15.4 network is combined with NB-IoT. To this end, new dual nodes are incorporated, acting as cluster heads. The paper discusses the different aspects of formation and operation of the combined network. A dynamic link selection (DLS) algorithm is also proposed, based on which cluster headers dynamically determine the preferred link, depending on link quality and type of traffic. Extensive simulations show that the DLS algorithm significantly increases battery life on dual nodes, which are the nodes with the highest power demands.

## 1. Introduction

Traditionally, communication has been understood as a type of human-to-human (H2H) relationship. However, the needs of industrial automation and information processing, together with advances in miniaturized electronics, have led to the consideration of a broader concept of relationships that includes machine-to-machine (M2M) communications. Subsequently, the Internet of Things (IoT) has evolved from the foundations established by M2M to allow communication between disparate devices and systems using Internet connectivity to marry different technologies and deliver interactive and fully integrated networks in different environments [1,2]. Wireless communication is one of the IoT enablers. The almost exponential growth in the number of wireless connections requires a rational use of radio resources. In the licensed spectrum, the fifth generation (5G) specification has defined the massive machine-type communication (MTC) service to face this huge increase in wireless networks [3,4,5], whose harmonization and optimization introduces major challenges [6,7]. Other types of networks complement the requirements of the IoT. Indeed, low data rate wireless personal area networks (LR-WPAN) are used in industrial control applications, smart cities, environmental monitoring, smart metering, transportation, or structural health.

The first technologies that implemented LR-WPANs (for example, Institute of Electrical and Electronic Engineers 802.15.4, Zigbee, Bluetooth Low Energy, DASH7, and WirelessHart) have been joined later by others with a wide range coverage and low power consumption, which are collectively called low-power wide-area networks (LPWANs). Among the LPWAN technologies we highlight the Long Range (LoRa) network, Sigfox, narrowband IoT (NB-IoT), and the Ingenu’s Random Phase Multiple Access (RPMA). While the next radio specification paves the way for 5G-based IoT applications, in practice it will coexist with the aforementioned LR-WPAN and LPWAN technologies. Therefore, it is necessary to design efficient mechanisms for their integration, so they can share resources and exchange information [8]. The interoperability between these networks can be addressed at the highest layers of the open systems interconnection (OSI) model, leading to an independent design and deployment of each of the networks. In this way, however, many of the possibilities offered by their combined use are wasted [9], limiting the size of the networks, increasing overall costs, and reducing the useful life of stand-alone terminals.

This paper presents the model of a combined wireless network using two different technologies: The Institute of Electrical and Electronic Engineers (IEEE) 802.15.4 standard and the NB1 or NB2 categories of the long-term evolution (LTE) specification, also known as NB-IoT. The design and deployment of the combined network is carried out jointly to take advantage of the specific characteristics of each of these two technologies. The simultaneous use of nodes with connectivity in only one of the networks, with others that have dual connectivity, allows the generation of a joint network that combines the global coverage of NB-IoT with the advantages of low consumption and low cost of LPWAN solutions. Reuse of existing networks is especially considered in our model, to take advantage of the fact that LR-WPAN technologies have been widely used in the past. Existing LR-WPAN nodes can be incorporated into the new network by using the mechanisms available for reconfiguration over the air (OTA). To define the new combined network, we begin by studying and characterizing the NB-IoT communication, taking into account those mechanisms intended for low power consumption: extended discontinuous reception (eDRX) and power save mode (PSM). Both have been directly inherited from LTE [10]. Discontinuous reception (DRX) already appears in Release (Rel.) 4, although it is not until Rel. 13 that the reception periods are extended to accommodate the requirements of the new LTE-NB1 category. Meanwhile, PSM mode is added for the first time in Rel. 12. The integration of the new NB-IoT nodes into an existing LR-WPAN and the implications they have on the resulting topology, formation, and routing algorithms are then studied.

The main contributions of this work are listed below:

- Study of possible LR-WPAN and LPWAN combination strategies in the context of IoT systems with low-power requirements;
- Definition of a cluster-tree topology for the combined network, where new nodes with double connectivity are added to an already existing IEEE 802.15.4x-based network. The design parameters in this case are the number of clusters, which is related to the total predictable population of the network, and the number of nodes per cluster;
- Proposal of a DLS algorithm in the cluster head (CH) nodes, which chooses between the two available links (IEEE 802.15.4 or NB-IoT) at runtime. This is a key feature of the proposed combined network.
- Run of validation simulations, which are carried out in an environment specifically designed for the characterization of the combined network, paying special attention to the incorporation of those nodes with an NB-IoT connectivity.

The rest of the paper follows this organization: the required background is reviewed in Section 2. Section 3 shows the state of the art in the design of combined wireless networks. Next, the combined network is described in Section 4 and simulation results that validate the advantages of the proposed solution are shown in Section 5. Finally, some conclusions are drawn in Section 6.

## 2. Background

### 2.1. From LR-WPANs to LPWANs

Wireless networks have evolved with the development of the IoT. Early wireless local area networks (WLANs), intended to replace the wired ones, were standardized in the late 1990s in the set of 802.11.X standards family from IEEE. These networks allow the exchange of data at high speed, up to 10 Gbps in the 802.11ax amendment (currently under development). However, their wide bandwidth (BW) makes the nodes of these networks have high hardware requirements and energy consumption, which are incompatible with current IoT applications that require simple, low-cost, battery-powered nodes with a low data rate. The development of wireless sensor networks, more adequate to the requirements of current IoT, began at the first decade of current century. Noteworthy is the work carried out by the IEEE 802.15 Working Group, which proposed a wide range of standards for different applications within industrial, scientific, and medical (ISM) unlicensed bands. In 2003, the first version of the IEEE 802.15.4 standard emerged, which defines the physical (PHY) and medium access control (MAC) layers for LR-WPANs. Network and higher layers have been standardized elsewhere, giving rise to well-known networks such as Zigbee, wireless highway addressable remote transducer (WirelessHART), and International Society of Automation (ISA) 100.11a. Among the technologies available for LR-WPAN not based on IEEE 802.15.4, we highlight Bluetooth low energy (BLE) and Z-Wave. New wireless technologies have recently appeared with the aim of increasing the link budget and further reducing the energy consumption. These LPWAN technologies seek to cover urban areas (up to 10–15 km of link range) with a simple network topology and a large population. The proprietary modulations of LoRa and SigFox, together with Ingenu’s proprietary access scheme RPMA, are some of the main exponents of these LPWANs in the unlicensed bands.

**Figure 1:**
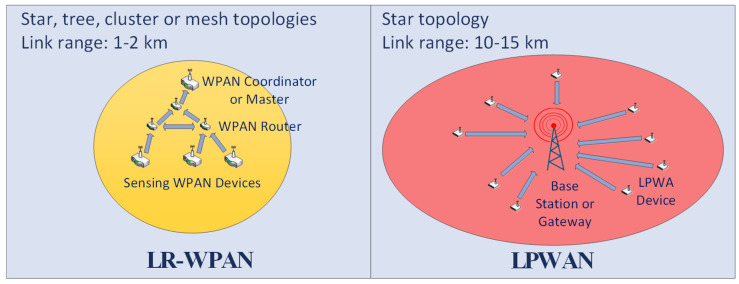
Network evolution from LR-WPANs to LPWANs.

Figure 1 depicts the evolution of wireless networks described in this section [9]. To extend the coverage with low energy consumption, LPWAN technologies employ two strategies [11,12,13]:

- Optimization of the modulation scheme. In practice, a choice is made between two different options: an extreme reduction of the BW by means of ultra-narrow band modulation (UNB), or the use of spread spectrum (SS). UNB modulation offers an increase in range thanks to the concentration of power in a narrow spectral band that leads to a reduction of the in-band noise at the receiver. Additionally, the ultra-high power spectral density of this scheme makes it resistant against interference and jamming. On the other hand, in the SS technique, the information is transmitted by alternating the pattern or frequency of the carrier, which allows a low power spectral density. The robustness against interferences and jamming is obtained in this case from its low spectral density and the need to know the spectrum spreading pattern at receiver side;
- Simplification of the network topology. Taking advantage of its extended coverage, LPWAN technologies use a star architecture, where a gateway or base station provides service to all devices that belong to its coverage area. Compared to the multi-hop configurations of the LR-WPANs, this simple topology avoids the relay of messages, which results in a simplification of the protocols and in reducing the overall energy consumption of the network.

In the case of the licensed bands, H2H broadband mobile communications have set the roadmap in LTE, universal mobile telecommunications system (UMTS), and earlier specifications. Starting with Rel. 13, new LTE categories for MTC (LTE-M) in IoT applications are defined in parallel. Third Generation Partnership Program (3GPP) dictated that a single transport technology does not fit all use cases, which represented a milestone for the arrival of the LPWAN approach to the mobile environment. 

With it, the LTE-M1 (BW: 1.4 MHz, bitrate: 1 Mbps) and LTE-NB1 (BW: 180 KHz, bitrate: 200 kbps) categories arose [14]. This idea is also maintained in the 5G specification, where smart mobile devices coexist with LPWAN devices using the same resources provided by the mobile network operator (MNO) for accessing mobile network [15].

Table 1 contains a simplified comparison of the main LPWAN technologies concerning spectral utilization, modulation technique, and other performance indexes, such as data rate, coverage, communication delay, and mobility [11,16,17,18]. Both licensed and unlicensed bands are covered.

### 2.2. Combining LR-WPAN and LPWAN Technologies

This paper aims to combine LR-WPAN and LPWAN technologies to take advantage of their individual benefits. According to [9], the integration can be undertaken at three different levels: (1) at the technology level, (2) at the device level, and (3) at the system level.

At the technology level, wireless networks can be combined at the PHY or MAC layers of the OSI stack. In the PHY layer the combination deals with the design of a transceiver capable of modulating/demodulating both LR-WPAN and LPWAN signals. Since there are no LR-WPAN technologies in the licensed band, only unlicensed LPWAN technologies are candidates. We mentioned earlier that most of the LPWANs in the non-licensed bands use two alternative approaches to modulate the signals in the PHY layer: SS and UNB, which allow to obtain the required characteristics, either by expanding the bandwidth in a controlled way or by narrowing the channel communication, respectively. LoRa and RPMA use the first strategy, while Sigfox uses the second one. A combined MAC protocol is also possible. For example, in each frame, the heterogeneous devices compete for any of the available time slots in the contention period. Only successful devices transmit their data in the time-divided sequence. If they have more packets in the buffer, they will reserve the same slots in subsequent frames. LR-WPAN and LPWAN devices must support the same MAC protocol during contention, with slotted ALOHA (S-ALOHA) and persistent or non-persistent carrier detection multiple access (CSMA) being the most common protocols [19,20,21].

At the device level, some (or all) nodes on the network contain two transceivers and can be connected through an LR-WPAN or LPWAN link, interchangeably. Additionally, packages can be shipped via both routes. In the design phase, the two transceivers can be included on one or two printed circuit boards. The same decision must be made about using one or two interconnected microcontroller units to control the transceivers. In theory, any of the LR-WPAN and LPWAN technologies could be combined at the device level, but coexistence and immunity to interference between the two technologies are mandatory. Dual connectivity allows:

- Definition of an efficient communication scheduling scheme;
- Optimization of the combined network in terms of power and reliability.

System-level combination is the most widespread option in the literature [11,22,23,24]. At the top layers, the combined solution consists of implementing LR-WPAN and LPWAN independently. The objective in this case is to guarantee interoperability between networks. The devices of a network communicate directly with other nodes of the same network and transmit the information to a central service from where the origin is discriminated and both networks are interconnected. The need to provide this service centrally is the main challenge for system-level networking. As an advantage, there are no changes in the lower-level protocols of each network. As a drawback, the advantages of a combined use of the technologies involved in each network are hardly taken advantage of.

## 3. Related Work

This section summarizes some of the most relevant work related to the combination of networks in the IoT. Here the heterogeneous, hybrid, and interoperable networks are discussed.

First of all, heterogeneity deals with the concurrent use of different radio access networks (RANs), as in [25]. Enhancements in deployment, interference mitigation, and resources utilization are pointed out as main research topics for heterogeneous cloud RANs [26,27]. As a matter of illustration, Wang et al. [28] make a deeper approach to the development of ultra-dense heterogeneous networks (HetNets). Although some authors also call heterogeneous networks those that integrate unlicensed band technologies (e.g., BLE [29] or Zigbee [30]), traditionally the term heterogeneity has been reserved for licensed in-band networks. Secondly, hybrid networks arise when wireless and high-performance wired systems are mixed. For example, a fiber optic distribution core network and some local wireless subnetworks covering small areas [31,32,33]. These fiber-wireless broadband access networks pursue an optimized and energy-efficient development of MAC and network layer protocols.

In [34], the authors identify some of the main implementation challenges for these kinds of networks, in relation to their integration into the low-power 5G ecosystem. They present a hybrid architecture with a next generation passive optical network with optical network units that are attached to 5G base stations. Another interesting hybrid network approach is found in [35]. A BLE network for smart cargo monitoring is proposed, where wireless devices send measurements from sensors to a gateway (GW) that contains a power line communications (PLC) adapter for communication with the central monitoring station through the PLC wired network. Finally, interoperability is related to the interconnection of different wireless systems on the upper layers of the IoT stack with a service-oriented architecture [11,22,23,24]. In this case, communications are not scheduled alternately and the ability to modulate data according to different schemes is not available. Authors in [11,36,37] apply interoperability between Zigbee and BLE networks, the Open Connectivity Foundation protocol, or IEEE 11073 standard, respectively.

In contrast, the concept of cooperative communication (CC) exploits the broadcast nature of wireless links, where multiple paths (in different technologies) are considered to send a packet. It has been seen as a promising technique for increasing the overall capacity of wireless systems, although relays must be controlled to achieve a measurable improvement over a direct link [38,39]. Jadoon and Kim [40] show an analytical model for CC exploiting the Q-learning reinforcement machine learning technique for optimal relay selection. Ge et al. [41] achieve cooperation by utilizing multiuser diversity, while preserving power allocation and resource sharing demands for every user. In this context, as indicated above, LoRa is one of the most widely employed LPWAN technologies in the unlicensed spectrum, and LoRa wide area network (LoRaWAN) is intrinsically cooperative on the UL [42,43]. It defines a star topology (see the LPWAN caption in Figure 1) where LoRa devices send information to the network server (NS) via one or more GWs. If several GWs receive data, they all transmit them to the NS. As explained before, a lower error rate and better performance can be achieved using this diversity because, although some GWs may be overloaded for the spreading factor (SF) employed by the transmitter, it is likely that some other GWs can transmit the information to the server without collisions. Even though, in LoRa specification, the NS selects one GW for DL transmission, a cooperative DL is also achievable [44]. It should be noted that cooperation in LoRa does not implement any link selection logic for UL enhancement, but rather all GWs in the coverage area receive data packets and, if no collisions occur, they retransmit them to the NS.

Networks combined at the technology or device levels can be found elsewhere in the literature. In [21,30], the time division multiple access (TDMA) sequence is scheduled to conform an efficient combined network based on LoRa and NB-IoT technologies, respectively. Garrido-Hidalgo et al. [29] develop a combined mesh network at the device level in the Industry 4.0 sector, where BLE and LoRa technologies are used for the acquisition of field data and context information.

Another device-level combination for two LR-WPAN technologies (ISA 100.11a and WirelessHart) is developed by Jecan et al. [45] for industrial environments. Dual connectivity LTE/5G and WLAN for mobile devices intended for personal use is also frequent in the literature [46,47,48,49]. This paper presents a combined network at the device level, with an architecture based on clusters, whose main differences with respect to other work in the bibliography are summarized in Table 2.

## 4. Efficient Design of the Combined Wireless Network (Based on NB-IoT and IEEE 802.15.4)

### 4.1. Network Design and Architecture

The advent of the IoT has led to the deployment of wireless networks with a large number of nodes. In many cases, these deployments are made to complement or extend existing LR-WPAN networks, which must be replaced by networks that use the new LPWAN standards or coexist with them. The cost of replacing the networks already deployed is very high, so the proposal of this work is to build a combined network that takes advantage of the best of both technologies. This is a common problem that, as an example, the authors of this work have found in updating and expanding wireless networks for monitoring and control in different industrial applications, such as: (1) railway traffic management in singular facilities [50], (2) the tracking of cargo containers in intermodal transport [51], (3) lighting and signaling of traffic intersections in rural areas [52,53], and (4) smart-metering in water distribution [54]. These applications have used different versions of the same LR-WPAN network based on the IEEE 802.15.4g standard. Following the standard nomenclature, each network contains a coordinator and several routers implemented with full function devices (FFDs), and up to 100 reduced function devices (RFDs), which act as end devices (EDs). Without loss of generality, we will use this LR-WPAN network as an example, when necessary.

#### 4.1.1. Description of the Existing LR-WPAN

Table 3 contains the most relevant features of the LR-WPAN used as an example in this study. An 802.15.4 beaconed network with a tree topology was chosen (see LR-WPAN caption in Figure 1), where the network coordinator serves as a link to the outside world through a GW. The depth of the tree was limited to four routing hops, in order to limit communication delay and energy consumption used in the relays. Devices at each level are dynamically identified through the 16-bit IEEE 802.15.4 short address, assigning a subset of addresses to each router.

Although any of the sub-GHz ISM bands of 169/434/868 MHz might be used, the 868 MHz band will be considered hereafter, as a trade-off between data rate and network coverage. Link budget reaches 141 dB, with a maximum distance between two nodes of up to 1800 m, in line of sight. The deep sleep characteristic of the transceivers used for the implementation of the nodes allows a consumption of only 4.9 μA during the inactive periods of the IEEE 802.15.4 superframe.

#### 4.1.2. Network Combination: Introducing Low-Power NB-IoT Links

The design of the combined network involves the making of a set of decisions, which are collected in Table 4. In our case, Step 1 was forced by the existence of a previous LR-WPAN deployment. From among the options considered in Section 2.2, a combined network was chosen at the device level, since does not require replacement of already deployed terminals. The main objectives pursued with the combined network are (1) expand the coverage of the original network to cover geographically dispersed areas, (2) minimize the cost of that transformation, and (3) improve the quality of service. An extended version of the sequence proposed in [9] to design a combined network is used in this paper.

NB-IoT is the chosen LPWAN technology. Since it belongs to a licensed band, coexistence with the LR-WPANs is ensured. Comparing to a technology in unlicensed band (LoRa/Sigfox/RPMA), the MNO provides the access network infrastructure and the evolved packet core (EPC). In addition, the quality of service (QoS) is also guaranteed by the MNO. When compared to LTE-M, the battery lifetime is extended by using a shorter bandwidth at the cost of lower transmission speed. Even so, the BW and data rate of NB-IoT are clearly higher than those provided by LoRa or Sigfox. It is noteworthy that the 3GPP guarantees compatibility between the different RANs, so that NB-IoT will be part of future 5G-based IoT networks. The most important characteristics of NB-IoT are listed in Table 1. It is relevant that, apart from the deployment within the LTE band that is used in LTE-M, narrowband NB-IoT carriers are allowed in two additional configurations: in the LTE guard band and isolated in the GSM band [55]. New narrowband channels are defined for NB-IoT [56]:

- Narrowband physical broadcast channel (NPBCH). It is a channel in the DL, where the messages are transmitted from the base station to all nodes with the generic information of the system.
- Narrowband physical DL shared channel (NPDSCH). It is a channel in the DL that contains the data transmitted from the network to one of the connected devices and the acknowledgments to the messages received by the UL.
- Narrowband physical UL shared channel (NPUSCH). It is a channel in the UL for the transmission of data and acknowledge messages from a device to the base station.
- Narrowband physical DL control channel (NPDCCH). It is a channel in the DL containing the control signals transmitted by the base station to establish the configuration for transmissions and receptions, as well as the number of repetitions.
- Narrowband physical random access channel (NPRACH). It is a channel in the UL for device access control. Random access (RA) procedure is initiated by sending a preamble [57].

The NPDSCH limits the size of the transmission blocks (TBS) based on the information shared in the information messages through the NPBCH and the NPDCCH. Specifically, the subframe index ($I_{SF}$) and the TBS index ($I_{TBS}$) establish a value of up to 680 bits for the LTE-NB1 category and up to 2536 bits for the LTE-NB2 category [57]. Similarly, in the case of NPUSCH, the maximum TBS coincides in the LTE-NB2 category while it is 1000 bits for the LTE-NB1 category. A repetition mechanism for the transmitted symbols is also established on all channels.

Repetitions are one of the two mechanisms used in LTE and NB-IoT to improve the probability of a correct reception of messages [57]. It involves repeating transmitted symbols a previously established number of times, so that the signal-to-noise ratio (SNR) at the receiver may be increased. Devices with poor connectivity, i.e., those that measure a low received reference signal power (RSRP), benefit from a high number of repetitions more than others. NB-IoT defines three coverage enhancement (CE) levels by repetitions: CE0, CE1 and CE2. The latter provides a link budget of 164 dB, up to 20 dB more than in legacy LTE. It is intended for distant or indoor located devices. Detailed descriptions of the repetitions mechanism in RA phase can be found in [58,59].

Up to 128 repetitions can be achieved in the UL, the maximum number is 2048 in the DL. The collision control procedure in NPRACH conforms to the media access implemented in NB-IoT. In massive environments, when several nodes transmit at the same time, collisions occur that are detected by each device when it does not receive acknowledgement (ACK). In this case, it waits for a random time and the same symbols are retransmitted taking into account the number of repetitions that has been established [6].

**Figure 2:**
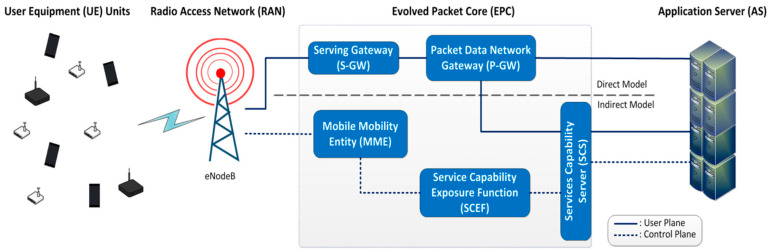
Simplified version of the radio access network (RAN) for NB-IoT UEs and the evolved packet core (EPC).

Figure 2 shows, in a simplified way, the access to the core of the mobile network through NB-IoT technology in the three possible configurations [60,61]:

- Direct model: direct communication through the user plane (UP), as in legacy LTE;
- Indirect model: connection through the utilities of the service capability server (SCS) to provide a better management of data interchanges over UP (lower power consumption) and to introduce communications over the control plane (CP);
- Hybrid model: simultaneous use of direct and indirect models.

**Figure 3:**
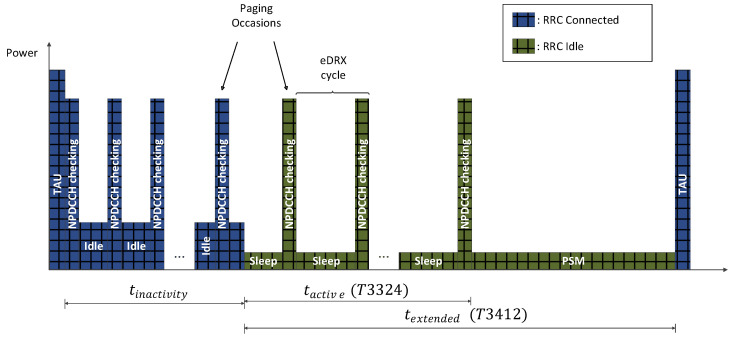
Paging cycles for a UE when eDRX feature is active.

Another important inheritance of the LTE standard has to do with the mechanisms for reducing the energy consumption of user equipment (UE). These are two optional functions, although highly recommended for reducing the consumption of network devices. On the one hand, eDRX consists of alternating brief periods in which the channel is actively listened for data reception (intervals with high power consumption) with intervals in which the UE does not attend possible receptions by monitoring the NPDCCH (intervals with low power consumption). This periodic procedure is called paging. For the sake of illustration, Figure 3 represents the UE power behavior between two consecutive tracking area updates (TAU) when no packets are transmitted between device and the evolved node base station (eNodeB). Typically, each paging cycle consists of one or more paging occasions followed by a sleep period, until the paging time window (PTW) is completed. This cyclical DRX behavior can occur while the device is in radio resource control (RRC) Connected or RRC Idle states. On the other hand, through the PSM mode the UE works in a very low consumption state where accessibility from the network is not available for sending or receiving messages. However, when leaving the PSM, no reassociation to the network is required since the registration information is preserved in the EPC and the device remains registered. It should be noted that this configuration can affect the latency of the communications on the DL, as the device does not receive any data while it is in PSM. It is the PSM which has the highest impact on the power consumption of the IoT network, being essential for the battery life objective of the UEs of more than 10 years with a 5 Wh battery, established by the 3GPP [62,63,64]. In addition to eDRX and PSM, the configuration of the NB-IoT link becomes relevant in the long-term consumption of the UE. Specifically, it is conditioned by the values of the following parameters: (1) inactivity timer, (2) extended timer, (3) active timer, (4) data rate, (5) number of UL/DL segments, (6) number of repetitions, and (7) modulation tones. More detail about the role of these configuration parameters can be found in [14,64,65].

#### 4.1.3. Proposed Architecture for the Combined Network

**Figure 4:**
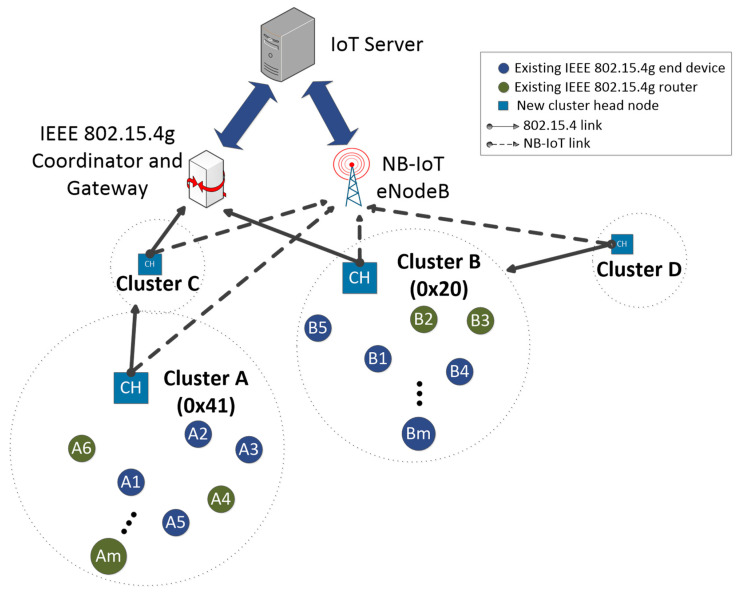
Network architecture with a cluster-tree topology [9].

The proposed combined network consists of the nodes of the original LR-WPAN and new devices with dual NB-IoT and IEEE 802.15.4g connectivity. These latest devices are named dual nodes. Combined network architecture at device-level is shown in Figure 4, where nodes are distributed into clusters and new dual nodes act as CHs. At any time, dual nodes can relay data as FFD routers of the 802.15.4 network or as data aggregators from the EDs of the cluster through NB-IoT, depending on which link is dynamically selected. Clusters are organized hierarchically, consisting of one CH and several EDs connected to it. Let us define m as the maximum number of EDs connected to a CH. A star topology is selected for each cluster, eliminating the original tree structure that allowed up to four hops. In this way, the number of retransmissions is reduced, the energy efficiency of the network increases and its latency is reduced.

Level 1 (L1) CHs communicate directly with network coordinator and can, in addition, relay information from level 2 (L2) CHs. In Figure 4, clusters B and C are L1, while A and D are L2 clusters. For simplicity, a CH node does not generate new information, just serving as a relay. This new structure makes the total number of routers decrease with respect to the original LR-WPAN. Old routers in the original LR-WPAN are reused as EDs. In addition, just by adding new CH nodes, EDs population in the combined network can increase with respect to the number of EDs in the original network.

Before booting the new combined network, an OTA firmware upgrade has to be made from the LR-WPAN GW, using the upgrade mechanism already existing in the old network. Once the software has been updated, the booting is carried out. To build the new combined network the nodes search for a parent node (a dual node acting as CH), using the same association protocol used in the original 802.15.4 network. Only dual nodes have a different initialization mechanism. Single nodes always act as EDs in the resultant network. Dual nodes can be preconfigured as L1 or L2 CHs in the network design phase. Nevertheless, final connectivity hierarchy depends on distance and link quality between nodes. L1 CHs search for a coordinator to associate with. When succeed, they can retransmit data through the two wireless interfaces. In the case they failed, they would become L2 CHs, which can communicate with other CHs and with the NB-IoT infrastructure. A CH located in an area with poor NB-IoT coverage will only employ the LR-WPAN link. Similarly, if any CH is not able to find a higher-level device (i.e., the GW or one L1 CH) for IEEE 802.15.4 association, then the only available link will be the LPWAN one. The next section gives more details on association and routing.

**Figure 5:**
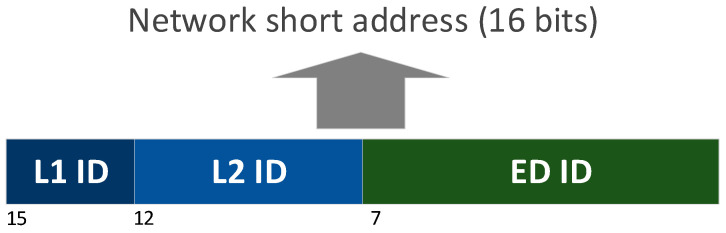
Addressing scheme of the combined network.

The addressing scheme in the combined network uses the IEEE 802.15.4 short addressing mode, taking advantage of the hierarchical structure of the clusters, so that the next hop on the network is determined without the need for a look-up table. Each cluster is identified through the 16-bit IEEE 802.15.4 short address of the corresponding CH. Figure 5 shows the methodology of address assignment by any node receiving an association request (i.e., CH or coordinator). There are three distinguishable fields:

- L1 ID (3 bits): identifies an L1 CH (and cluster). Coordinator assigns to each L1 CH the first available position in this field. The rest of the bits are 0.
- L2 ID (5 bits): used to identify an L2 CH (and cluster). Parent CH gives the corresponding address using this field. The remaining bits match.
- ED ID (8 bits): identifies each ED inside a cluster. CH utilizes this field for each associated node. The most significant byte is not altered.

The operation of each of these fields is similar. The first associated node receives the value 1, second node the value 2, and so on. For example, Table 5 lists the cluster identifiers and short addresses of some of the nodes in Figure 4. Let us suppose that the CH of cluster C receives a message whose destination address is 0x4105. Since it does not belong to any ED in its cluster, it proceeds to reroute the message. To do this, it applies the mask 0xFF00 to the destination address and retransmits the message to the resulting address (that is, 0x4100, which is the address of the CH of cluster A). As stated before, the role of the nodes in the original LR-WPAN may be modified in the combined network.

Let us assume that the original LR-WPAN network had $N_{T}^{\prime }$ number of nodes: $N_{R_{T}}^{\prime }$ routers, $N_{ED}^{\prime }$ EDs, and 1 network coordinator. In the combined network, let $N_{T}$ be the number of nodes, γ the fraction of the original network routers which are not reused, and $N_{ED}$ the number of new EDs. Once the maximum ED population per cluster is defined (i.e., m parameter), the number of clusters ($N_{Cl}$), the original network node replacement factor, η, and the density of reused nodes, δ, are calculated as:

(1) $$N_{Cl}=\;\lceil \frac{N_{T}}{m+1}\rceil =\lceil \frac{\left(1-\gamma \right)N_{R_{T}}^{\prime }+N_{ED}^{\prime }+N_{ED}}{m}\rceil ;$$

(2) $$\eta =\frac{\left(1-\gamma \right)N_{R_{T}}^{\prime }+N_{ED}^{\prime }+1}{N_{T}^{\prime }},\;\delta =\eta \frac{N_{T}^{\prime }}{N_{T}}.$$

Expression (1) reflects that, in the case that the network population was the maximum allowed, the clusters would be balanced. Note that $N_{Cl}$ parameter strongly influences the efficiency of the cluster formation and information forwarding algorithm, as it is affected by the size of the network [66]. η and δ determine the degree of reutilization of the LR-WPAN hardware and they depend on the type of IoT application for which the combined network is designed. They are metrics that allow the comparison of different combined networks in terms of deployment. One of the advantages of the proposed combined network is the ease of its deployment. In principle, only the new CH nodes need to be added to the existing network, and its number is clearly small when compared to the total number of nodes. For the number and location of these CHs, different approaches can be carried out (e.g., they could replace the routers of the old network). A more sophisticated approach would consist in the selection of new, optimized locations by using network planification; this optimization would take into account the location of the network nodes, the type of traffic and the LPWAN coverage, in order to balance the communication burden between the CHs and extend battery lifetime. This will be the topic of future research.

### 4.2. Routing Algorithm and Dynamic Link Selection

Hierarchical path selection mechanisms allow to control the overall energy consumption versus flat arrangements where all nodes share the same unchangeable role [67]. Both grid layout and clustered organization are employed for this purpose [66,68]. In this work we employ the cluster-based architecture defined above. A description of common cluster formation and routing algorithms is found in [67,69,70]. Referring to the configurability of clusters, Table 6 shows the parameters used by the combined wireless network that directly or indirectly affect the procedure for generating and operating them [71]. All cluster-based routing algorithms involve a two-step operation. Initially, cluster formation consists of electing CH nodes for every cluster in the network and of permitting the association of EDs. Then, a steady state phase is reached when all active nodes belong to the hierarchical network. EDs transmit and receive data on demand or periodically. CHs relay information through the preferred communication link. DLS manages the necessary switches of the CH preferred link. Routing algorithm is simplified in the case of the combined network for different reasons:

- It is not necessary to implement any CH selection phase, since all dual nodes that are added to the network acquire that role. The selection of CHs implies delays and higher consumption in the initialization of the network.
- Parameter *m* is predefined at system startup, although it can be subsequently modified from the server at runtime. Therefore, the number of clusters is preassigned.
- Network consists of static nodes, facilitating the management of communications within and between clusters.
- Previous router nodes change their role and become EDs, sending data to IoT server.
- For the formation of clusters, LR-WPAN decision-making is used in the phase of association of a device to the network. The best CH is selected in terms of link quality, number of nodes connected to it, and received signal power.

The initial network configuration supports two possibilities by activating one of the following preprocessing directives:

- *RANDOM_SETUP*: each CH randomly chooses the IEEE 802.15.4 or NB-IoT link for sending UL messages.
- *PREDEFINED_SETUP*: the preferred link to be chosen by each CH is assigned in code, considering its location or availability. Network designer must decide in each individual case.

At initialization, active L1 CHs perform passive frequency scanning in the 868 MHz band and, upon detection of the LR-WPAN coordinator, associate to it. Belonging to L1 implies that any coordinator is supposed to be found. If it was not possible, received beacons from other active L1 CHs would be analyzed. A new L2 hierarchy role is assumed by those L1 CHs that are not able to connect to coordinator. They select a best candidate and send the association request. When no eligible candidates exist for a CH, it initiates a waiting interval and keeps trying until that timer expires. Unsuccessfully associated CHs get a reduced functional role as single nodes. Once the LR-WPAN startup ends, the initialization of the LPWAN interface takes place. Subsequently, the CH activates the NB-IoT transceiver for registration to the mobile network. Again, the single role functionality would be acquired if there was not enough NB-IoT coverage. Active L2 CHs follow an analogous procedure, except for the coordinator-seeking state. EDs receive beacons from all active CHs in range, choosing the best candidate in terms of detected power levels. If a CH reaches the maximum of m EDs connected to it, it stops accepting more children.

In this way, all routes between devices are established. The next step is the preferred link selection for each CH among the available IEEE 802.15.4 and NB-IoT links. For example, assume that the CH in cluster C (Figure 4) selects the NB-IoT link. Henceforth, it will group data from EDs connected to its cluster and cluster A. Then, the CH forwards it to eNodeB in as few messages as possible. The unpicked link is set in a low-power state: (1) deep sleep mode for IEEE 802.15.4, and (2) 413 days and 0 s values of the extended and active timers, respectively, for NB-IoT.

With the wireless network formed and active, the proposed DLS algorithm is run on each CH node and allows runtime modification of the preferred communication link among the two transceivers. Any link switch leads to activation of the new preferred link and switching of the other link to low-power state. The change of link decision can be made by the CH itself or by any node with a higher hierarchy role, as detailed below.

A link switch directive is given in the following situations:

- From CH:
  ○ If a single packet exceeds the fixed maximum number of retransmissions for each link: *MAX_RTX_IEEE* and *MAX_RTX_NBIOT*. Note that when it is exceeded on NB-IoT link, it is not switched to a higher CE level. The preferred link is switched, and the message is transmitted over IEEE 802.15.4.
  ○ If the average received power level is less than a certain amount (*PWR_THR_IEEE* and *PWR_THR_NBIOT*) in five consecutive transmissions.
  ○ If connectivity to parent CH on IEEE 802.15.4 link is lost.
- From parent CH:
  ○ If any L1 CH retransmits data from a very large number of children (greater than *MAX_CHILDS*), it can send a link switch message, *LINK_SWITCH*, to an L2 CH from among its children to switch to the NB-IoT link. Note that for each low-hierarchy cluster, assuming it takes *L* bits to format data in an ED, up to *m* × *L* additional bits are transmitted each time.
- From GW or server:
  ○ If the maximum number of retransmissions since last link change in the global set of packets transmitted by a CH is reached, GW will trigger a *LINK_SWITCH* request to that particular CH.

Link switching control parameters are determined according to the IoT solution for which the combined network is designed.

For example, in a smart metering application the number of CHs located indoors (at a CE level higher than that of the outdoor nodes), represents a compromise between the repetitions established in the preferred NB-IoT link and the number of link changes that are produced. This count of link switches occurring in the combined network reflects the congestion and reduction of communication quality situations that took place since network startup.

## 5. Results

This section presents the results obtained for the proposed combined network concerning energy consumption and reliability. The NS-3 simulator was used. Based on C++ and Python programming languages, it allows to characterize the temporal behavior of the nodes of a network distributed in a specific geographical area and with pre-established message exchange requirements. NS-3 allows the selection of multiple communication protocols and support extensions for the implementation of new technologies or modification of existing PHY and MAC layers.

In the scope of this paper, the “lte” module is modified to comply with the NB-IoT standard, while the “lr-wpan” module is used as the basis for the IEEE 802.15.4 links of the combined network. Simulations allow us to characterize the behavior of the presented model and to evaluate the proposed DLS algorithm applied to CHs. In total, 500 iterations over the interval of interest are performed through Monte Carlo simulations. Time range of simulations extends over 1 year of network operation or until the CH battery level falls below a given threshold. As in the original IEEE 802.15.4 network, the nodes that support the highest energy expenditure are those that retransmit the messages from/to the EDs due to the need to complete the processes of receiving individual messages, grouping, and sending packets towards the eNodeB or LR-WPAN coordinator. Therefore, the graphs presented here refer to the CH nodes with the highest battery consumption.

Let us consider a combined network composed of five clusters (three CHs acquire an L1 hierarchy role, the rest are L2 CHs). One of the main variables under study is $N_{T}$, analyzing its effects on power consumption and reliability. Nodes are placed at random positions. They are powered on during an initial interval of 3 min.

**Figure 6:**
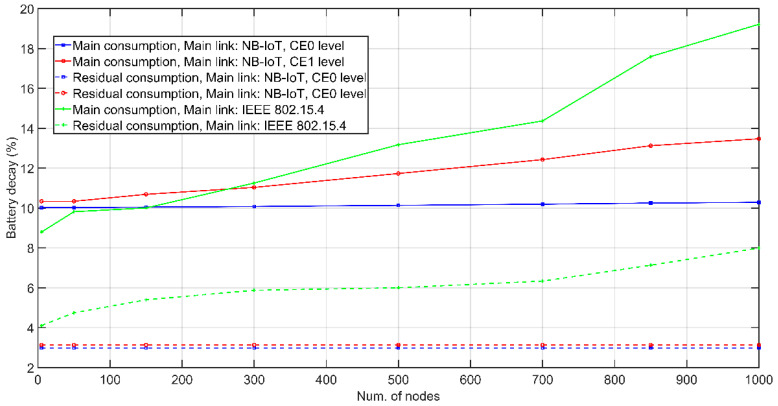
Annual battery decay of the combined network as a function of the number of nodes.

Unless otherwise indicated, each ED transmits a message with a payload of 6 B per day, collecting measurements of some magnitude of interest. Added to this are the network management and control messages that are exchanged in the DL. Table 7 indicates NB-IoT setup when it is utilized as the preferred link. Remember that UE and eNodeB negotiate main parameters. As established by 3GPP, the initial battery capacity of every node equals 5 Wh [57]. Power consumption input data, experimentally assessed, for IEEE 802.15.4 and NB-IoT transceivers are displayed in Table 3 and Table 8, respectively. Battery discharge suffered by a CH in a period of 1 year is represented in Figure 6 for the different contributions: (1) communications over main link and over secondary link when NB-IoT link is chosen, and (2) communications over main link and secondary link when IEEE 802.15.4 is selected as preferred. Consider that one of the CHs at each level is assigned at the highest level of coverage improvement, CE1, while the others belong to level CE0. By having a higher number of repetitions configured, at the CE1 level, the battery is depleted more pronounced with the increase in the number of segments in the UL. Shown graphs refer to the L1 CH and the L2 CH with the highest battery consumption, i.e., the worst case.

**Figure 7:**
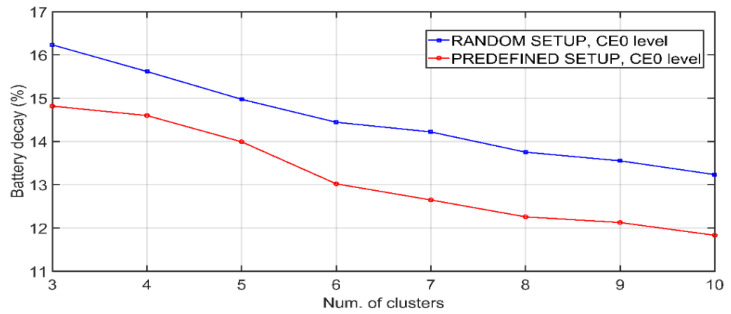
Annual battery decay of the combined network as a function of the number of clusters.

**Figure 8:**
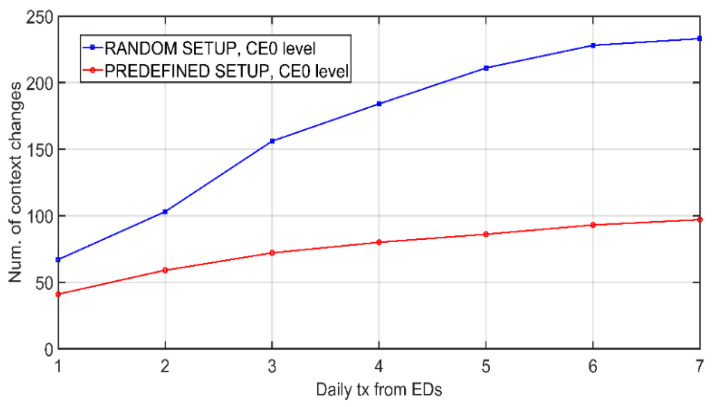
Number of link switches as a function of daily data rate.

**Figure 9:**
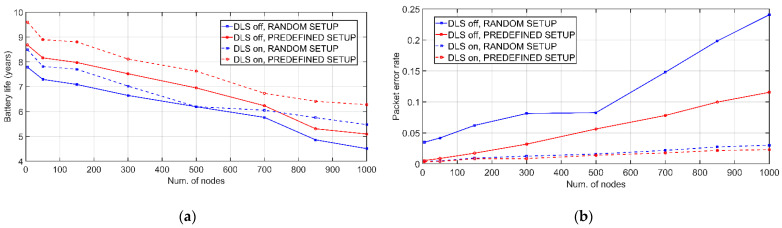
Effects of DLS algorithm on the combined network as a function of network population: (**a**) battery lifetime; (**b**) packet error rate.

In the case of Figure 7, battery level is shown as a function of the number of clusters in the network for the same operating interval. Network population is 500 nodes. As there are fewer clusters, *m* becomes larger, so that the battery is consumed more. The *RANDOM_SETUP* and *PREDEFINED_SETUP* settings are evaluated. This second case requires a prior analysis of each link to choose the one with the best connectivity, requiring measurements prior to the system’s startup. In this way, a better efficiency in battery consumption is achieved. Next, the effects of the DLS algorithm are analyzed. Figure 8 shows the effect of the number of daily transmissions from EDs on link switches produced by DLS algorithm. As the exchanges increase, more retransmissions and preferential link switches occur. CE0 level nodes are more prone to runtime link switches due to the lower number of retransmissions in both UL and DL. Additionally, Figure 9 elaborates on the effects of DLS algorithm on the combined network durability and error rate. The dependency of battery lifetime on the total number of nodes in the network is plotted in Figure 9a. Each simulation runs until the first CH depletes the battery. For the case of illustration, with a total network population of 1000 nodes, the application of the DLS algorithm translates into an average of 49 annual link changes for the L1 CH with *PREDE-FINED_SETUP* and 101 changes assuming that the initialization is *RANDOM_SETUP*. Again, it is observed that the random start configuration produces a more pronounced energy waste. Furthermore, in approximately 97% of the cases, DLS algorithm yields an improvement in battery life.

For densely populated networks, the increase is more than 20% of the value obtained without applying dynamic selection. By the way, Figure 9b shows packet error rate under the same assumptions as above. This algorithm makes the errors smaller and minimizes the influence of network population on the messages not properly received. Regarding the combined network validation, no works have been found using the same two technologies with a joint approach. Furthermore, the high simulated times (6 months, 1 year or several years; depending on the circumstances) make the results promising. The combined network enables system scalability by directly obtaining networks with a higher number of EDs than the deployed IEEE 802.15.4 networks.

Compared to a deployment only based on NB-IoT, the combined network solution has the following advantages:

- Lower installation cost;
- Reutilization of the previous network infrastructure;
- Hierarchical architecture;
- Provision of coverage in areas with poor NB-IoT connectivity;
- Possible lower delays, for non-delay tolerant IoT applications.

## 6. Conclusions

This paper deals with combining existing LR-WPANs and new LPWANs to meet some common challenges in wireless communication networks, such as scalability, geographic coverage, reliability, and QoS. This type of combination also prepares the integration of existing networks in the 5G standard. The proposed procedure consists of six sequential design phases: (1) choice of LR-WPAN technology, (2) choice of LPWAN technology, (3) choice of the combination level, (4) design of the combined network architecture, (5) network configuration and, optionally, (6) network training. In this paper, an IEEE 802.15.4 network, already deployed in different scenarios, was chosen to be combined with NB-IoT. The new combined network has a hierarchical architecture based on clusters, whose CH are new dual devices that have double connectivity (802.15.4 and NB-IoT). These nodes accept the connection of EDs and relay the messages, either through the 802.15.4 coordinator, if available, or through the NB-IoT EPC. During the network creation, the preferred link for dual nodes is set by default or randomly. A DLS algorithm is defined to reduce power consumption and extend the battery life of CHs. Simulations were carried out for a network with up to five clusters, organized in two levels, and a variable number of nodes (up to 1000) covering a 1 year time. In each case, 500 iterations were carried out, varying the initial configurations according to a Monte Carlo model. The results show that the useful life of the network (i.e., the time elapsed until the battery of the first dual node is exhausted) can be extended by more than 20% using DLS technology. The method proposed here can be applied, with appropriate modifications, to another set of combined LR-WPAN and LPWAN networks, providing a solution for the integration of pre-existing networks into the IoT of the future. For example, the utilization of LoRa (unlicensed spectrum) and LTE-M (for non-static applications) will be addressed in a future combined network. Meanwhile, a real testbed is being deployed to measure power consumption and error rate. Finally, future work comprises new strategies for optimized location of the CHs.

## Figures and Tables

**Table 1:** Main features of LPWAN relevant technologies (UL: uplink, DL: downlink).

Technology	LoRa	SigFox	RPMA	LTE-M	NB-IoT
Spectrum	Unlicensed	Unlicensed	Unlicensed	Licensed	Licensed
Frequency band	Sub-1GHz ISM	Sub-1GHz ISM	2.4GHz ISM	LTE bands: 1, 2, 3, 4, 5, 7, 8, 11, 12, 13, 18, 19, 20, 25, 26, 27, 28, 31, 39, 40, 41	LTE bands: 1, 2, 3, 4, 5, 8, 11, 12, 13, 14, 17, 18, 19, 20, 25, 26, 28, 31, 66, 70, 71
Modulation	Proprietary chirp SS (CSS)	UL: UNB DBPSK DL: UNB GFSK	UL: Proprietary RPMA-DSSS DL: CDMA	UL: SC-FDMA DL: OFDMA	UL: SC-FDMA DL: OFDMA
BW	Configurable (<500 kHz)	Europe: 100 Hz USA: 600 Hz	1 MHz	1.08 MHz	180 kHz
Data rate	Up to 50 kbps	Europe: 100 bps USA: 600 bps	Up to 624 kpbs	Up to 375 kbps	DL: Up to 226.7 kbps UL: Up to 250 kbps
Range	5–10 km	10–20 km	10 km	10 km	15 km
Latency	10 ms	30 ms	10 s	10 s	10 s
Mobility	Yes	Yes	No	Yes (handover)	Intracell

**Table 2:** Comparison between this work and other terminology in the literature.

Concept	References	Type	Differences
Heterogeneous Networks	[25,26,27,28]	Wireless, only mobile technologies	This article considers dual-connectivity: NB-IoT (licensed band) and IEEE 802.15.4-based (unlicensed band)
Hybrid Networks	[31,32,33,34,35]	Mixed: wired and wireless	This article is focused exclusively on wireless technologies
Interoperable Networks	[11,22,23,24,36,37]	Wireless	This article proposes the combination of two wireless technologies in the link or network layer of the OSI model

**Table 3:** Features of the deployed IEEE 802.15.4-based LR-WPANs [9].

Item	Value	
PHY/MAC Protocol	IEEE 802.15.4g	
Frequency band	Sub-GHz ISM (868 MHz)	
Operation	Beaconed	
Topology	Tree	
Depth	Four levels	
Power supply	3.8 V battery (per node)	
Power consumption	Idle: 79.2 mW Sleep: 16.17 µW	Tx: 462 mW Rx: 211.2 mW
Network population	Up to 120 nodes	
Link budget	141 dB	
Max. link coverage	1800 m	
Data rate	Up to 200 kbps	
Other features	Deep sleep	

**Table 4:** Decision-making in the combined network design sequence.

Step	Description	Decision
1	Selection of LR-WPAN technology	IEEE 802.15.4
2	Selection of LPWAN technology	NB-IoT
3	Selection of integration degree	Device-level
4	Design of the combine solution	Dual connectivity Cluster-tree topology
5	Network setup	OTA firmware update, if required Routing algorithm
6	Network learning	Link selection

**Table 5:** Cluster identifiers and CH addresses from Figure 6 scheme.

Cluster	Level	L1 Bits	L2 Bits	Id	CH Address	Parent Cluster	Some EDs Addresses
A	2	010	00001	0x41	0x4100	0x40	A1: 0x4101 A5: 0x4105
B	1	001	00000	0x20	0x2000	-	B3: 0x2003 B4: 0x2004
C	1	010	00000	0x40	0x4000	-	C2: 0x4002 C26: 0x401A
D	2	001	00001	0x21	0x2100	0x20	D6: 0x2106 D9: 0x2109

**Table 6:** Configuration of clustering main parameters in the context of the combined network.

Parameter	Description	Application to Combined Network
CH selection	Election algorithm (deterministic or stochastic)	Not necessary (preassigned CHs)
Cluster formation	Methodology for node acceptance into clusters	Distributed approach (IEEE 802.15.4g)
Types of nodes	Classification in terms of node capabilities	Heterogeneous nodes: CHs and EDs are different (hardware and software)
Mobility	Dynamic handover within clusters	Static nodes
Levels of hierarchy	Description of the hierarchical architecture	Three-level hierarchy: L1 CHs, L2 CHs, and EDs

**Table 7:** Configuration of the preferred NB-IoT link.

Parameter		Value	
Extended timer		12.5 days	
Active timer		1 min	
CE levels (repetitions)	CE0	UL: 2 repetitions	DL: 4 repetitions
	CE1	UL: 24 repetitions	DL: 64 repetitions
UL transm. block duration		10 ms	
Windows per eDRX cycle		4	

**Table 8:** Energy consumption on NB-IoT link.

State	Power Consumption
Idle	21 mW
Transmission	716 mW
Reception	213 mW
PSM	15 μW

## Data Availability

NS-3 code of the modified modules (lte and lr-wpan) is available under request by email to the corresponding author: jpgarcia@gie.us.es. Further details on how to get started with NS-3 programming can be found online at https://www.nsnam.org/docs/release/3.33/tutorial/singlehtml/index.html (accessed on 26 May 2021).
